# Construction of a biocompatible MWCNTs–chitosan composite interface and its application to impedance cytosensing of osteoblastic MC3T3-E1 cells

**DOI:** 10.1039/d2ra05995a

**Published:** 2022-11-04

**Authors:** Jun Zhong, Jing Huang, Liang Chen, Jiang Duan

**Affiliations:** Department of Orthopedics, Renmin Hospital of Wuhan University Zhangzhidong Street 9 Wuhan 430060 People's Republic of China zhongjun8496@aliyun.com; Key Laboratory of Pesticide & Chemical Biology of Ministry of Education, Central China Normal University Wuhan 430079 China

## Abstract

In this work a carboxylated MWCNTs–chitosan composite sol–gel material was developed *via* one-step electrodeposition on a glassy carbon electrode as the cytosensing interface of a novel impedance cytosensor. SEM verified the formation of a three-dimensional hierarchical and porous microstructure favorable for the adhesion and spreading of osteoblastic MC3T3-E1 cells. By correlating impedance measurements with fluorescence microscopic characterization results, the cytosensor was demonstrated to have the ability to determine the MC3T3-E1 cell concentration ranging from 5 × 10^3^ to 5 × 10^8^ cell per mL with a detection limit of 1.8 × 10^3^ cell per mL. The impedance cytosensor also enabled monitoring of the cell behavior regarding the processes of cell attachment, spreading, and proliferation in a label-free and quantitative manner. By taking advantage of this cytosensing method, investigating the effect of the C-terminal pentapeptide of osteogenic growth peptide (OGP(10–14)) on MC3T3-E1 cells was accomplished, demonstrating the potential for the application of OGP(10–14) in bone repair and regeneration. Therefore, this work afforded a convenient impedimetric strategy for osteoblastic cell counting and response monitoring that would be useful in evaluating the interactions between osteoblastic cells and specified drugs.

## Abstract 2

A composite interface enables the impedance cytosensing of osteoblastic MC3T3-E1 cells for counting cells, monitoring cell spreading and proliferation as well as the interactive response to an osteogenic growth peptide active component.

## Graphical abstract

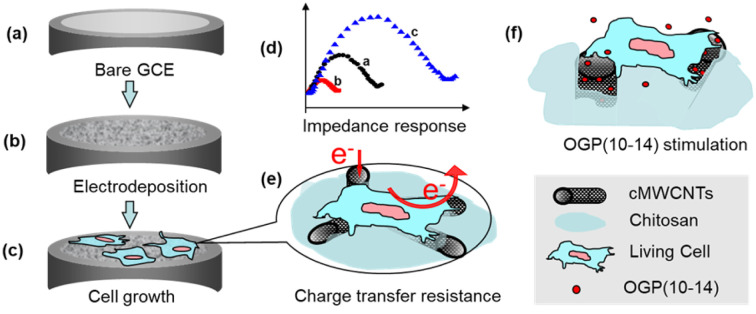

## 1 Introduction

Cell impedance biosensors are a type of electrochemical device^1^ that employs living cells on the electrode surface as a sensing component to detect cell responses to various stimuli by measuring the impedance variation.^2^ With the ability to analyze the biological events of cell adhesion, spreading, proliferation, and apoptosis and other processes in a label-free and quantitative fashion, cell impedance biosensors have the potential to be used as powerful tools in an array of fields such as drug screening, toxicology testing, and cytophysiological and pathological mechanism research.^3^ The characteristics of electrode interface play a crucial role in successful applications since the interface needs to provide not only a microenvironment for accommodating the living cells but also an electrochemical connection to the electrode for electric signal transduction.^2^ Therefore, developing new electrode interfacial construction strategies by using optimal materials or material combination have been the research focus of cell impedance biosensors to achieve desired performance.

To date, various materials have been reported for the interface construction of cell impedance biosensors, such as metallic and carbon-based nanoparticles,^4^ synthetic^5^ and bionic polymers,^6^ quantum dots,^7^*etc.* Chitosan and its modified versions^8^ and multi-walled carbon nanotubes (MWCNTs)^9^ are among the most widely employed materials for cytosensing interface construction. Chitosan is a biocompatible and biodegradable natural polymer with a flexible molecular backbone and numerous amino and hydroxyl groups that render chitosan soluble in acidic solution and can be exploited for further functionalization. While MWCNTs are electricity-conductive, biocompatible, relatively rigid, and under acidic conditions can be dispersed in the solution of chitosan to form a well homogeneous state. These characteristics of chitosan and MWCNTs make them suitable partners for cytosensing interface construction.^10^ The carboxylated version, cMWCNTs, which is more hydrophilic and dispersible, has been also employed for this purpose.^11^ In addition, silica sol–gels, which are formed by *in situ* hydrolysis of various silicates such as tetraethyl orthosilicate under acidic or basic conditions, has been considered a kind of glue for building stable biosensing interfaces due to their ability to form covalent three-dimensional networks with other components,^12,13^ showing promising potential in constructing various biosensor interfaces. Nevertheless, the combination of the specified three materials has not been reported thus far for constructing a cytosensor interface.

On the other hand, murine osteoblastic MC3T3-E1 cells are a type of model cell line that has been utilized for investigating its interactions with a variety of scaffold materials and evaluating the role of the interfacial morphology and osteogenic growth factors.^14^ Osteogenic growth peptide (OGP), along with its active form C-terminal pentapeptide OGP(10–14), is a class of biologically active peptide hormones with promising effects on the proliferation and differentiation activity of osteoblastic MC3T3-E1 cells.^15^ Recently, using OGP-based supramolecular hydrogels to promote hBMSC osteogenesis differentiation^16^ and OGP-functionalized highly porous scaffolds to stimulate the proliferation and growth of osteoblastic cells^17^ were also disclosed, suggesting these OGP-based scaffolds have promising potential as biomaterials for bone regeneration. Despite these important results, there are no reports regarding cytosensing investigation of the MC3T3-E1 cell behavior and the effect of (OGP)/OGP(10–14) on the cell adhesion, spreading, and proliferation, and these knowledges would be helpful to comprehension of osteoblastic cell–material interactions and development of novel cytosensing interface materials.

**Scheme 1:**
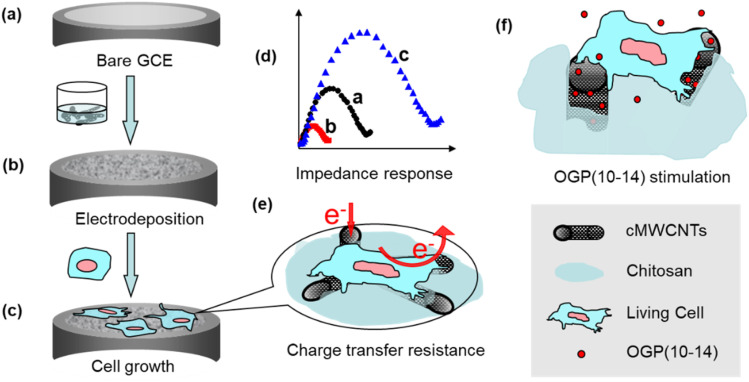
Schematic of interface construction and impedance cytosensing: (a) bare GCE; (b) electrodeposition of the cMWCNTs–chitosan composite sol–gel; (c) adhesion and spreading of MC3T3-E1 cells; (d) impedance signals at the three stages of (a) to (c); (e) charge transfer resistance by the cells; (f) stimulating MC3T3-E1 cell with OGP(10–14).

Given the advances of impedance cytosensors and their interface materials, in the present work, a novel impedance cytosensor for osteoblastic MC3T3-E1 cells was constructed by a one-step electrodeposition of three sol–gel components, chitosan, carboxylated MWCNTs, and a silica precursor (3-aminopropyl)triethoxysilane (APTES), on glassy carbon electrode (GCE). This type of composite sol–gel cytosensing interface was highly porous and cytocompatible, hence suitable for MC3T3-E1 cell growth and electrochemical impedance monitoring. By correlating impedance measurements with fluorescence microscopic characterizations, the impedance cytosensor was demonstrated to be very useful in counting MC3T3-E1 cells and monitoring cell growth and stimulus response to OGP(10–14) (Scheme 1).

## 2 Materials and methods

### 2.1 Reagents

MWCNTs with purity of above 95%, length of about 50 μm, and diameter in 20–30 nm were purchased from Shenzhen Nano-Harbor Corporation (China) Ltd cMWCNTs were obtained by carboxyl functionalization with a mixture of 65% HNO_3_ and 98% H_2_SO_4_ (1 : 3, v/v) for 6 h under sonication.^11^ OGP(10–14), the OGP active C-terminal pentapeptide with the fragmental sequence of (NH_2_-YGFGG-OH),^14^ was custom-synthesized by GL Biochem (Shanghai) Ltd. The MC3T3-E1 mouse osteoblast cell line was purchased from Type Culture Collection Center of Chinese Academy of Sciences (Shanghai, China). Cell staining dyes, 4′,6-diamidino-2-phenylindole (DAPI) and 3,3′-dioctadecyloxacarbocyanine perchlorate (DIO), were purchased from Biosharp Ltd (Korea). Silicate coupling agent (3-aminopropyl)triethoxysilane (APTES), chitosan (95% deacetylation), phosphate buffer solution (PBS) and other reagents were of analytical grade and used as received. Aqueous solutions were prepared with double-distilled water.

### 2.2 GCE modification with cMWCNTs–chitosan composit sol–gel

Glassy carbon electrodes (GCE) with 3 mm in diameter were polished successively with 1.0, 0.3, and 0.05 μm alumina slurry, followed by rinsing and sonicating with double-distilled water and alcohol, and dried at room temperature. To prepare the cMWCNTs–chitosan composite sol–gel solution, a chitosan acetic acid solution (0.5% chitosan at pH 5) was first made; then to this solution proper amounts of cMWCNTs and water were added to give a cMWCNTs-dispersed solution with the cMWCNTs content of 1.0 mg mL^−1^ and chitosan concentration of 0.05%; and finally the composite sol–gel solution was obtained by mixing 2300 μL of the chitosan acetic acid solution, 600 μL of cMWCNTs-dispersed solution, 60 μL of APTES, and 40 μL of 30% H_2_O_2_ solution. Electrodeposition was performed using a CHI660D electrochemical workstation (Shanghai Chenhua, China) and a three-electrode system with a saturated calomel electrode (SCE) as the reference electrode, a platinum foil as the counter electrode, and the cleaned GCE as the working electrode. The composite sol–gel solution was used as the electrolyte, and the electrodeposition was conducted using a constant potential at −0.4 V *vs.* SCE and room temperature for 300 s. The modified electrodes were carefully rinsed with water, then dried for 60 min in air and stored at 4 °C for use. The morphology of the modified electrode interface was characterized by SEM (JEOL-6700F, Japan).

### 2.3 Cell culture

The osteoblastic MC3T3-E1 cells were cultured at 37 °C in DMEM medium (Gibco, Life Technologies) in a humidified atmosphere containing 5% CO_2_. The DMEM medium was complemented with 10% fetal calf serum (FBS) (by volume, Tianjin Haoyang Biological Corporation, Ltd China), 100 μg mL^−1^ penicillin, and 100 μg mL^−1^ streptomycin (Gibco, Life Technologies). Then the cells were digested with 1.5 mL of 0.25% trypsinase (Gibco, Life Technologies), separated from the medium by centrifugation at 1000 rpm for 5 min, and washed twice with a sterile PBS at pH 7.4. The cells were diluted with serum-contained medium to yield a cell suspension as stock solution with a final concentration of 5 × 10^8^ cells mL^−1^. Cell suspensions with various concentrations were prepared from this stock. For the cell culture on the electrode surface, 5 μL of the cell suspension was dropped on the electrode and incubated at 37 °C for a certain time. OGP(10–14) immobilized GCE was prepared by dropping 5 μL of the OGP(10–14) solution on the cMWNTs–chitosan composite sol–gel surface, maintained at a humidified atmosphere overnight for cell culture. The cell immobilized GCE was rinsed thoroughly with PBS three times for electrochemical impedance measurements.

### 2.4 Electrochemical measurements

Cyclic voltametric measurements were performed in pH 7.4 PBS on the CHI660D electrochemical workstation and the three-electrode system as described in electrodeposition. Electrochemical impedance spectroscopy (EIS) was carried out on an Autolab PGSTAT30 workstation (The Netherlands) using a 0.01 M pH 7.4 PBS solution containing 5 mM Fe(CN)_6_^3−/4−^ (1 : 1 in molar ratio) with 1.0 M KCl as the supporting electrolyte, The amplitude of the applied sine wave potential was set at 5.0 mV within the frequency range of 0.1 Hz–100 kHz. All measurements were carried out at 37 ± 2 °C.

### 2.5 Inverted fluorescence microscopy

For inverted fluorescence microscopy characterization, glassy carbon sheets instead of GCE electrodes were used, and the surface modification and MC3T3-E1 cell culture were conducted using the same procedures mentioned above. The cells were washed three times with pH 7.4 PBS solution, followed by incubation in 4% glutaraldehyde in 0.1 M sodium cacodylate buffer for 30 min at room temperature. Then the samples were washed with PBS and stained with 5 μg mL^−1^ staining dye DAPI or DIO for 30 min at room temperature. After washed with PBS solution, the samples were dried in air for observation. Fluorescence images were acquired using LEICA DMI 3000B inverted fluorescence microscope (Leica Corp., Germany). Control experiments were carried on bare glassy carbon sheets.

## 3 Results and discussion

### 3.1 Preparation and characterization of cMWCNTs–chitosan composite sol–gel interface

Organic–inorganic sol–gels are extensively used for constructing biosensors to improve stability and biocompatibility of the interface structure and to enhance the capability of accommodating biological species,^18^ owing to their desired advantages such as tunable porosity, chemical inertness, and film-forming ability.^19^ In the present work, three materials, chitosan, cMWCNTs, and the silicate precursor APTES, were combined to form the sol–gel solution. To accelerate the formation of the cytosensing interface film on the electrode surface, a proper amount of H_2_O_2_ was added and electrochemically reduced at a potential of −0.4 V *vs.* SCE to hydroxide, which raises the local pH, leading chitosan from soluble to insoluble state^19^ and to crosslinking with APTES to give a stable and rigid composite sol–gel film.

**Fig. 1:**
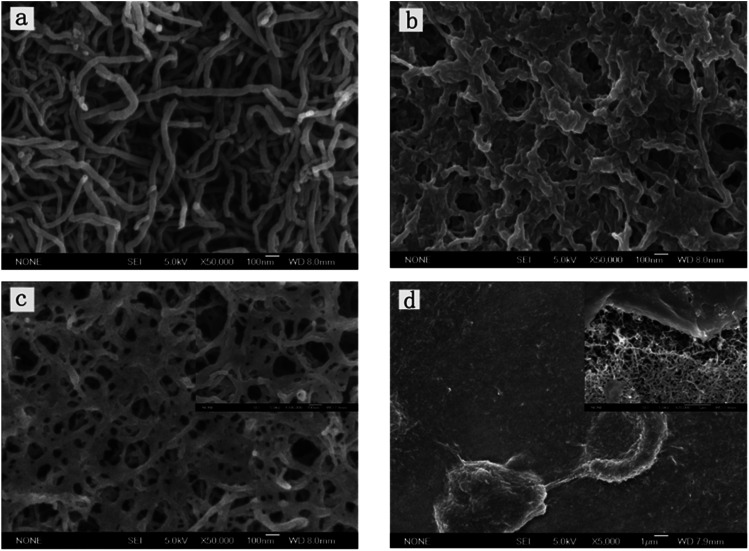
.SEM images: (a) cMWCNTs; (b) cMWCNTs–chitosan mixture; (c) cMWCNTs–chitosan composite sol–gel film; and (d) MC3T3-E1 cells on the composite film (cultured for 3 days). Scale bars = 100 nm (a–c) and 1 μm (d). Inserted images by magnification of 100 000× (c) and 20 000× (d) with bar scales of 100 nm and 1 μm, respectively.

SEM images in Fig. 1a–c show plainly the difference of the morphologies of cMWCNTs, cMWCNTs–chitosan mixture, and cMWCNTs–chitosan composite sol–gel film. The pristine cMWCNTs with diameter of 20–30 nm are worm-like discrete nanotubes (Fig. 1a), which become a coagulate state after treatment with a 0.05% chitosan aqueous solution with acidity adjusted to pH 5.1 by adding a concentrated NaOH solution (Fig. 1b), due to the insolubility of chitosan after deprotonation.^20^ Under the electrodeposition conditions, the raised local pH at the electrode interface resulted in the formation of chitosan-wrapped cMWCNTs and thus a highly porous morphology of cMWCNTs–chitosan composite sol–gel with cMWCNTs in diameter ranging from 20 to 30 nm is presented. The porous microstructure is of vital importance for cell survival because it can serve to mimic the actual *in vivo* microenvironment where cells interact and growth and to facilitate nutrient and oxygen diffusion and waste removal.^21^

Osteoblastic cell responses to the nano-micro multiscale cMWCNTs–chitosan composite sol–gel film was also assessed using SEM to characterize the cell morphology when cultured after 3 days on the film surface. As shown in Fig. 1d, the cells spread out and cover the surface well. Two cells, one with flat round shape and another with elongated shape, form connection with each other. These facts demonstrate that the cells response to and interact with the composite sol–gel film properly,^22^ reflecting the as-prepared film is cytocompatible and favorable to the attachment and growth of osteoblastic MC3T3-E1 cells.

### 3.2 Electrochemical characterization of MC3T3-E1 cells at the electrode interface

**Fig. 2:**
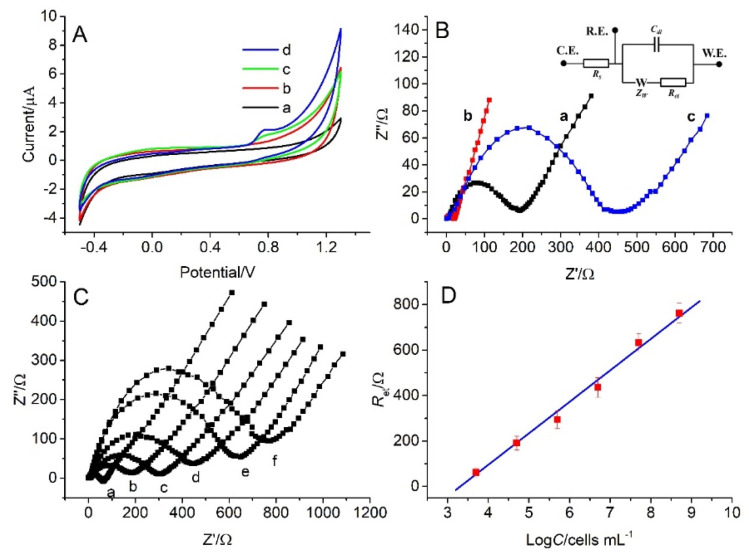
.(A) Cyclic voltammograms of (a) bare GCE, (b) composite film/GCE, (c) MC3T3-E1 cells/GCE, and (d) MC3T3-E1 cells/composite film/GCE in pH 7.4 PBS. (B) Impedance spectra ranging from 0.1 to 10^6^ Hz in 1.0 M KCl with [Fe(CN)_6_]^3−/4−^ as the redox probe for (a) bare GCE, (b) composite film/GCE, (c) MC3T3-E1 cells/composite film/GCE with the equivalent circuit model shown as inset. (C) Nyquist diagrams of composite film/GCE after incubation for 12 h in MC3T3-E1 cell suspensions with concentration of (a) 5.0 × 10^3^, (b) 5.0 × 10^4^, (c) 5.0 × 10^5^, (d) 5.0 × 10^6^, (e) 5.0 × 10^7^, (f) 5.0 × 10^8^ cells mL^−1^. (D) Linear plot of *R*_et_*versus* logarithm of cell concentration (log *C*) in the range from 5.0 × 10^3^ to 5.0 × 10^8^ cells mL^−1^ with a LOD of 1.8 × 10^3^ cells mL^−1^.

Metabolism processes within live cells involve a variety of redox reactions and changes of ionic composition and concentration, producing detectable electrochemical signals on a suitably modified electrode for characterizing cell activity.^23^ The cyclic voltammograms were acquired in pH 7.4 PBS for bare GCE, cMWCNTs–chitosan composite film/GCE, MC3T3-E1 cells/GCE, and MC3T3-E1 cells/composite film/GCE as shown in Fig. 2A.

The bare GCE has a minimum background current, which is raised in the high potential region of 0.6–1.3 V on the other three electrodes, attributable to the presence of chitosan and MC3T3-E1 cells that form larger double layer capacitances in this region on the respective surfaces (Fig. 2A, curves a–d). Compared with the direct attachment of the cells with GCE, which displays only a raised capacitance current (curve c), the cells on the electrode with the cMWCNTs–chitosan composite film present an irreversible oxidation peak at +0.78 V *vs.* SCE in the voltammogram (curve d). A similar observation was reported on a gold nanoparticles-chitosan nanocomposite modified GCE where an oxidation peak at nearly the same potential position was produced from the K562 leukemia cell response. The oxidation peak was ascribed to the detectable electron transfer in the intracellular conversion of guanine to 8-oxo-guanine in the living cells.^24,25^ In our case, the oxidation peak at +0.78 V *vs.* SCE might also come from the same intracellular reaction because like gold nanoparticles the cMWCNT conductivity could mediate the charge transfer within the cells to the electrode surface, making the intracellular reaction detectable. The output of the electrochemical signal suggests that the cells are maintained in a living state and the composite interface provides a compatible environment for the cells.

The significant influence of the cells on the electrode electrochemical process comes from the impedance characterization, from which the electron transfer resistance (*R*_et_) of the cell adhesion on GCE was measured using [Fe(CN)_6_]^3−/4−^ as the redox probe. Fig. 2B shows the impedance spectra of bare GCE (a), cMWCNTs–chitosan composite film/GCE (b), and osteoblastic MC3T3-E1 cells/cMWCNTs–chitosan composite film/GCE (c), obtained by fitting the data with the inserted equivalent circuit model. The curves (a) and (c) exhibit typic impedance semicircles from which the respective *R*_et_ values are estimated as 197.2 ± 15.7 Ω and 450.7 ± 27.4 Ω. While the cMWNTs–chitosan composite modified GCE shows the response as a nearly straight line, with a *R*_et_ value as low as 19.7 ± 2.5 Ω, indicating that the composite film provides a desirable pathway for the interfacial electron transfer with almost no resistance to the redox process of the [Fe(CN)_6_]^3−/4−^ probe. On the other hand, the osteoblastic MC3T3-E1 cells attached on the composite film form a barrier to electron transfer, resulting in the significant increase of *R*_et_ value to 450.7 ± 27.4 Ω (curve c). In addition, these interfaces show negligible solution contact resistance *R*_s_, which was also observed in the graphene oxide quantum dots/cMWCTs composite modified electrodes,^11^ favorable to the interface charge transfer.

The impedance response of live cells on electrode surface has been exploited for constructing sensitive and label-free cytosensors for many applications such as drug screening, toxicology testing, and cytophysiological and pathological mechanism research.^3^ In the present work, the relationship between the cell concentration and the impedance *R*_et_ value was also investigated. Fig. 2C shows the Nyquist diagrams of the composite film/GCE after incubation for 12 h in the osteoblastic MC3T3-E1 cell suspensions with successively increased concentrations from 5.0 × 10^3^ to 5.0 × 10^8^ cells mL^−1^. A linear relationship between *R*_et_ and the logarithm of cell concentration (log *C*) can be established as a linear regression equation *R*_et_ (Ω) = 142.07 log *C* − 484.25 with a correlation coefficient of 0.9912 and a limit of detection (LOD) of 1.8 × 10^3^ cells mL^−1^ as estimated from 3 times standard deviation of *R*_et_ value (Fig. 2D). According to this linear relationship the cell density in a MC3T3-E1 cell culturing suspension could be easily determined, affording a simple and straightforward way for the cell number quantification.

### 3.3 Monitoring of MC3T3-E1 cell behaviour on cMWCNTs–chitosan composite film/GCE

**Fig. 3:**
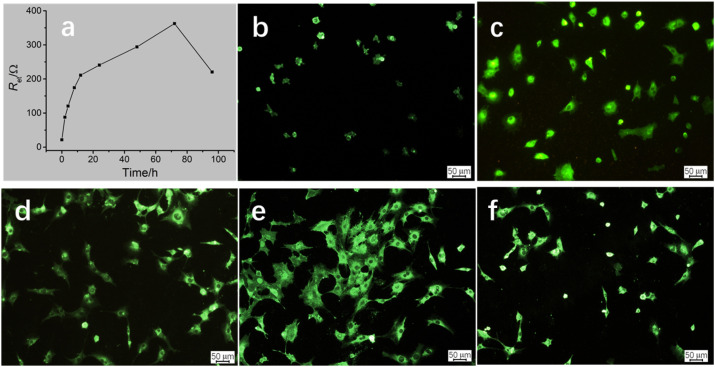
.(a) Variation of *R*_et_ with incubating time of MC3T3-E1 cells on the composite film/GCE after incubation for 0 h, 2 h, 4 h, 8 h, 12 h, 24 h, 48 h, 72 h, and 96 h. Fluorescence images (100×) of cells on the composite film coated glassy sheets at (b) 2 h, (c) 6 h, (d) 12 h, (e) 72 h, and (f) 96 h stained by DIO.

On the basis of the established cMWCNTs–chitosan composite film/GCE cytobiosensor and the linear relationship between *R*_et_ and log *C*, forthcoming investigation was focused on the MC3T3-E1 cell behavior on the composite film/GCE surface, and understanding the cell growth process would be helpful to the application in investigating the interactions of cell factors and drugs with the cells.^26^ The cells were incubated on the cMWCNTs–chitosan composite modified electrodes at 37 °C and the impedance response was monitored. Fig. 3a displays the time course of the *R*_et_ value variation with incubating time. The *R*_et_ value increases rapidly before the incubating time of 12 h, and then the increase rate becomes slow but steady till the time reaches 72 h, after which the *R*_et_ value drops speedily. To correlate the impedance response with cell behavior, inverted fluorescence microscopy was applied, and the fluorescence images corresponding to the incubating times are given in Fig. 3b–f. It can be seen that, at the incubating time 2 h, the cells began to attach to the surface; at 6 h, more cells inhabited on the surface with cell occupying area slightly enlarged; at 12 h, the cells became larger and the cell number somewhat also increased; at 72 h, the cells spread substantially and accompanied with the still increase in cell number; and finally at 96 h, the cell number on the surface was notably decreased and cell contraction was observed for the attached cells.

Careful analysis of the variation trends of the *R*_et_ value and the cell behavior on the composite/GCE surface, a preliminarily correlation could be drawn between the two variation trends, that is, the *R*_et_ value is closed related to the number and occupying area of the attached cells. The cell number provides a main contribution to the *R*_et_ value, which is significant before the incubating time 12 h, resulting from the cell attachment plus probably the cell proliferation. The MC3T3 cell proliferation in this time scale was also observed for *in vitro* cultured on polyaniline doped titania nanotubes biointerface.^27^ The cell spreading process contributes less to the *R*_et_ value, which mainly occurs in the incubating time ranging from 12 h to 72 h, because in this stage the cell spreading is predominant.

### 3.4 Role of OGP(10–14) on MC3T3-E1 cells

**Fig. 4:**
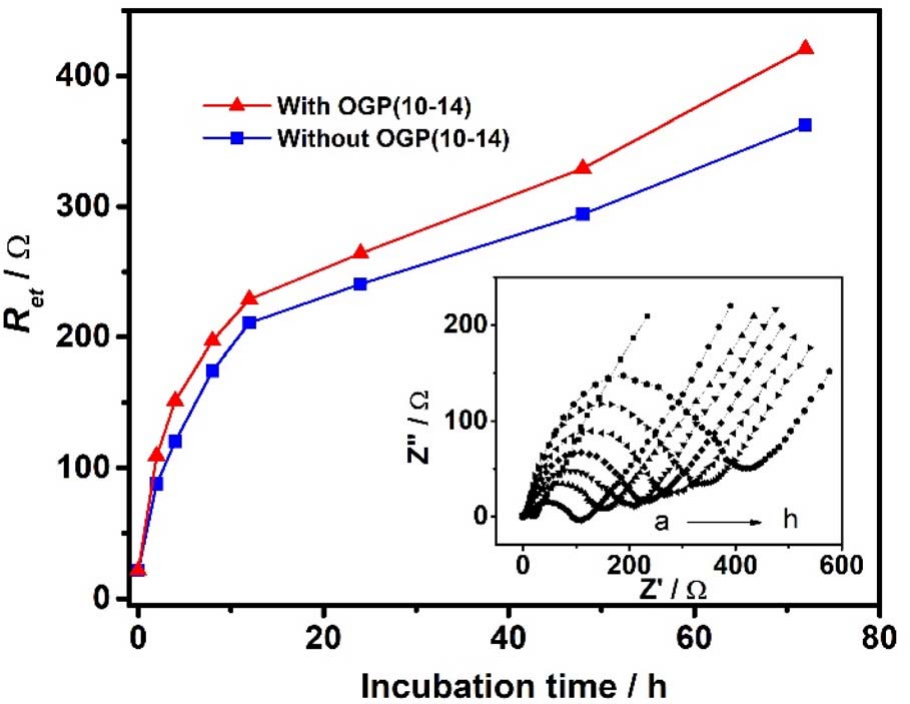
.Impedance responses of osteoblastic MC3T3-E1 cells on the cMWCNTs–chitosan composite film/GCE surface after incubation in solutions containing 1 × 10^5^ cell per mL of the cells with the presence (red curve) and absence (blue curve) of 10^−12^ M OGP(10–14) for (a) 0 h, (b) 2 h, (c) 4 h, (d) 8 h, (e) 12 h, (f) 24 h, (g) 48 h, and (h) 72 h. Inset: corresponding EIS spectra of osteoblastic MC3T3-E1 cells in the presence of 10^−12^ M OGP(10–14) for the corresponding incubation times.

As another potential application, the fabricated cMWCNTs–chitosan composite film/GCE cytosensor was used to investigate the role of OGP(10–14) by impedance monitoring the behavior of MC3T3-E1 cells. OGP(10–14), as well as OGP, has the potential in medical applications for bone repair and regeneration through stimulating the proliferation and growth of osteoblast cells, as demonstrated by administration or incorporation into scaffolds composed of various materials such as poly(lactic-*co*-glycolic acid),^28^ poly(ester urea),^29^ and chitosan-coated poly(lactic acid).^17^ However, to our best knowledge, there are no reports on the role of OGP and OGP(10–14) using cytosensing methodologies. Fig. 4 shows the impedance responses of MC3T3-E1 cells on the sensor surface after incubation in solutions containing 1 × 10^5^ cell per mL of the cells with the presence (red curve) and absence (blue curve) of 10^−12^ M OGP(10–14) in a period of 72 h. Again, the *R*_et_ value increases rapidly before the incubating time of 12 h, and then the increase rate becomes slow but steady, and at any inspection time point, the presence of OGP(10–14) results in a larger *R*_et_ value, corresponding a higher density of cell, than that without OGP(10–14), suggesting the promoting role of OGP(10–14) on the cell attachment, spreading, and proliferation on the cMWCNTs–chitosan composite film/GCE cytosensing surface.

**Fig. 5:**
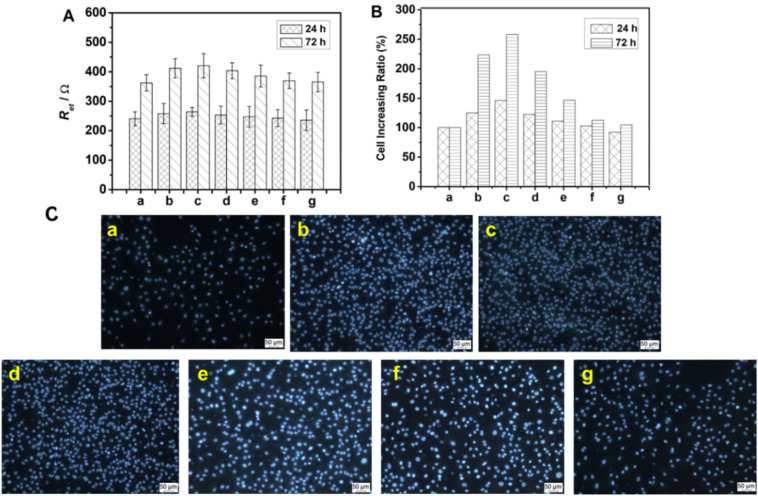
.Impedance responses (A) and cell increase percentages (B) of MC3T3-E1 cells after incubating 24 h and 72 h in the presence of OGP(10–14) at concentrations of (a) 0, (b) 10^−13^, (c) 10^−12^, (d) 10^−11^, (e) 10^−10^, (f) 10^−9^, (g) 10^−8^ mol L^−1^, and inverted fluorescence images (×100) (C) of cells after incubation with the same OGP concentrations for 72 h stained by DAPI.

The cytosensor was also used to determine the concentration-dependent effect of OGP(10–14) on the osteoblastic MC3T3-E1 cell behavior, in couple with the characterization by inverted fluorescence microscopy. At varying OGP(10–14) concentrations of 0, 10^−13^, 10^−12^, 10^−11^, 10^−10^, 10^−9^, and 10^−8^ mol L^−1^, the corresponding *R*_et_ values after incubation for 12 h and 72 h were measured, and the results are shown in bar graph in Fig. 5A. For 12 h incubation the variation of OGP(10–14) concentration has no significant influence on the impedance response because the *R*_et_ values are only slightly different; whereas for 72 h incubation, the *R*_et_ value varies more significantly, with the largest response centered at the OGP(10–14) concentration of 10^−12^ mol L^−1^. From these data, an optimal OGP(10–14) concentration of 10^−12^ M was identified, which produced the maximum *R*_et_ increases of 9.73% (*P* < 0.05) and 16.15% (*P* < 0.05) after 24 h and 72 h incubation, respectively. To further illustrate the OGP(10–14) effect, by calculating with the linear relationship established in Fig. 2C, the cell number increase in percentage relative to that without OGP(10–14) after incubation for 12 h and 72 h are plotted in bar graph as shown in Fig. 5B. At the optimal OGP(10–14) concentration of 10^−12^ M, the cell number increase in percentage reached 46.12% and 158.09%, respectively, after 24 h and 72 h incubation. Higher or lower concentration could reduce the effect of OGP(10–14). This result is consistent with observation of the OGP(10–14) effect on primary human osteoblast culture that resulted in a bell-shaped proliferation curve centered at the OGP(10–14) concentration of 10^−12^ M, due to the autocrine/paracrine mode of endogenous OGP in the cells regulated by exogenous OGP(10–14).^30^

The bell-shaped curve of impedance response with stimulation of different OGP(10–14) concentrations can be confirmed by and correlated to the fluorescence observation of the cell density variation after incubation with OGP(10–14) in the same concentration range (0–10^−8^ mol L^−1^), as shown in Fig. 5C. At the OGP(10–14) concentration of 10^−12^ mol L^−1^, the most densely populated cells cover the entire surface (Image c in Fig. 5C), higher than the cell coverage at any suboptimal or supraoptimal OGP(10–14) concentration. The results suggest the reliability of the cMWCNTs–chitosan composite sol–gel as the cytosensing interface for investigating the drug-stimulated cell behavior.

## 4 Conclusions

Cell impedance biosensors have become a powerful tool to analyze the biological events of cell adhesion, spreading, proliferation, apoptosis, and other processes in a label-free and quantitative fashion, finding potential applications in the fields of drug screening, toxicology testing, *etc.* However, for fabricating an osteoblastic cell cytosensor, developing a suitable electrode interface material is still challenging and remains to be explored due to the propensity of osteoblastic cells and the associated environmental requirements.

In this work an osteoblastic cell-compatible and highly porous cMWCNTs–chitosan composite sol–gel material was developed and used as the cytosensing interface material *via* a one-step electrodeposition on the GCE surface as a novel cytosensor. SEM verified the three-dimensional hierarchical and porous microstructure favorable to the adhesion and spreading of cells. The cytosensor was used for determining osteoblastic MC3T3-E1 cell concentration and thus a linear relationship was established in the range from 5 × 10^3^ to 5 × 10^8^ cell per mL with a detection limit of 1.8 × 10^3^ cell per mL. The impedance cytosensor was able to reveal convincingly the cell behavior regarding the cell attachment, spreading. And proliferation. Furthermore, the effect of OGP(10–14) on the osteoblastic MC3T3-E1 cells was investigated using this impedance cytosensor, showing the potential of OGP(10–14) in bone repair and regeneration. Therefore, this work established a novel impedimetric cytosensing strategy for osteoblastic cell concentration determination and stimulus response monitoring that would be very useful in estimating the interactions between osteoblastic cells and interfacial materials or relevant drugs.

## Author contributions

Jun Zhong: conceptualization, writing – review & editing, funding acquisition. Jing Huang: investigation, resources, writing – original draft. Liang Chen: formal analysis, data curation. Jiang Duan: supervision, formal analysis, project administration.

## Conflicts of interest

There are no conflicts to declare.

## Supplementary Material
